# Protocadherin FAT1-associated membranous nephropathy after hematopoietic stem cell transplantation—a cohort study with clinico-pathological correlations

**DOI:** 10.1093/ckj/sfag005

**Published:** 2026-01-10

**Authors:** Maja C Nackenhorst, Daniela Milovanovic, Alice Schmidt, Nicola Mongera, Maximilian Köller, Werner Passler, Daniel Cejka, Nicolas Kozakowski

**Affiliations:** Department of Pathology, Medical University of Vienna, Vienna, Austria; Department of Pathology, Medical University of Vienna, Vienna, Austria; Division of Nephrology and Dialysis, Department of Medicine III, Medical University of Vienna, Vienna, Austria; Nephrology Department, Bolzano Hospital, Italy; Department of Pathology, Medical University of Vienna, Vienna, Austria; Nephrology Department, Bolzano Hospital, Italy; Department of Nephrology, Hypertension, Transplant Medicine, Rheumatology, Geriatrics, Ordensklinikum Linz Elisabethinen, Linz, Austria; Department of Pathology, Medical University of Vienna, Vienna, Austria

**Keywords:** FAT atypical cadherin 1, formalin-fixed, hematopoietic stem cell transplantation, immunofluorescence, membranous nephropathy, paraffin-embedded

## Abstract

**Background:**

The identification of causative antigens for membranous nephropathy (MN) has revolutionized the comprehension of the pathophysiology of this autoimmune disease and its management. Recently, some studies described protocadherin FAT1 (FAT1) as a causal antigen in the context of hematopoietic stem cell transplantation (HSCT). The aim of this study was to investigate the large Viennese kidney biopsy collection at the Department of Pathology of the Medical University of Vienna and explore MN cases after HSCT for the causative antigen.

**Methods:**

Fifteen patients were identified with MN in diagnostic renal biopsies for proteinuria/nephrotic syndrome after HSCT between 2001 and 2024. Cases were reviewed and tested for the expression of FAT1 by immunofluorescence specifically.

**Results:**

Immunofluorescence showed that 12 out of 15 patients with MN had FAT1 expression, appearing 2.3 years after HSCT. In three patients without FAT1 expression, MN appeared 3.8 years after HSCT, with one testing positive for phospholipase A2 receptor (PLA2R) and two with unknown antigens. Serum creatinine for FAT1+ MN was 1.2 mg/dl, while FAT1− MN was 1.3 mg/dl. Proteinuria was 8 g/g compared to 10.4 g/g, respectively. IgG4 positivity was seen in 30% of FAT1+ MN cases. Over a follow up of 5.2 years, of the seven FAT1+ MN patients treated with rituximab, four achieved complete remission, two partial remission, and one was non-responsive.

**Conclusion:**

This hitherto largest single-center-cohort solidifies FAT1 as a causative antigen for MN in the setting of HSCT. However, not all post-HSCT-MN are related to FAT1.

KEY LEARNING POINTS **What was known:**

- FAT1 was recently described as a target antigen in patients with MN after HSCT.
- A cohort of 14 FAT1-positive MN from two North-American centers was originally described.
- Little is known about the course of the disease.

**This study adds:**

- FAT1 can be reliably identified using immunofluorescence performed on FFPE material.
- The hitherto largest single-center study of patients with MN after HSCT.
- Important clinico-pathological correlations, especially in regards to therapy.

**Potential impact:**

- Solidifies FAT1 as a target antigen in MN after HSCT.
- Provides a reliable protocol for immunofluorescence-FAT1 detection on FFPE material.
- Sheds light on possible therapeutic approaches for FAT1+ MN after HSCT.

## Background

Membranous nephropathy (MN) is an autoimmune disease characterized by the subepithelial deposition of immune complexes along the glomerular capillary walls. Its course remains unpredictable, with spontaneous remission rates of around one-third, one-third of patients responding well to immunosuppression, and one-third evolution to end-stage kidney disease [1]. The discovery of phospholipase A2 receptor (PLA2R) as a target antigen in MN improved the possibilities for disease detection and surveillance [2]. Novel detection methods such as laser microdissection in combination with tandem mass spectrometry (MS/MS), allowed the further identification of neural epidermal growth factor-like 1 protein (NELL1), semaphorin 3B (SEMA3B), protocadherin 7 (PCDH7), and serine protease HTRA1 in primary MN, exostosin 1/exostosin 2 (EXT1/EXT2) and neural cell adhesion molecule (NCAM1) in other forms of MN [3–8]. Multiple recent reports on MN of previously unknown etiology in the setting of hematopoietic stem cell transplantation (HSCT) identified protocadherin FAT1 (FAT1) as a likely target antigen, which was also observed in the context of antibody-mediated rejection and *de novo* MN [9–13].

We identified 15 patients with MN after HSCT in the Viennese kidney biopsy data bank and explored the expression of the antigen FAT1 via immunofluorescence from the formalin-fixed paraffin embedded tissue (FFPE) of the corresponding biopsies. We provide the hitherto largest single-center-cohort with clinico-pathological correlations.

## Materials and methods

### Patients and biopsies

We screened our local kidney biopsies data base for patients with MN after HSCT diagnosed at the Department of Pathology at the Medical University of Vienna (MUV) between 1997 and 2024. All patients underwent at least one renal biopsy due to proteinuria/nephrotic syndrome after HSCT and were diagnosed with MN via light microscopy and wherever possible confirmed with electron microscopy (14 out of 19 biopsies). Two patients underwent two biopsies each (patient 2, 4 years apart; and patient 3, 3.5 months apart). One patient underwent five biopsies (patient 9, over the course of 20 years): he received a renal transplant 6 years after the first biopsy displaying post-HSCT MN, which eventually recurred in the transplant 3.5 years later (in the meantime there had been one implantation biopsy and a diagnostic post-transplant biopsy without MN, which were not included in the report focusing exclusively on biopsies with post-HSCT MN). The diagnosis of MN was re-evaluated by two pathologists (M.N. and N.K.) in all cases. Four control patients with MN were included: two with PLA2R-positive MN and two with lupus nephritis class V according to the ISN/RPS classification. Data are provided as the median (interquartile range) throughout this paper.

### Clinical parameters

We performed a retrospective search in our in-house database to identify all patients with MN after HSCT diagnosed by kidney biopsy. From all 15 patients identified, we evaluated creatinine values, eGFR, and proteinuria at time of presentation and at 1-year, 3-year, and 5-year follow up, whenever available. We also noted therapeutic strategies and date of last follow up or death. The study was conducted according to the principles of the Declaration of Helsinki. Ethical approval was obtained from the MUV (No. 1589/2020) and the Kepler University of Linz (No. 1261/2024). The requirement for informed consent was waived due to the retrospective nature of the study.

### Histology and electron microscopy

All cases were cut in serial sections of 2–3 µm and stained with hematoxylin-eosin (H&E), periodic acid-Schiff, acid fuchsin orange G (AFOG), and methenamine silver according to in-house protocols.

Routine electron microscopy examination was performed on glutaraldehyde fixed samples kept in a cacodylate buffer solution until processing, consisting of a staining procedure with 1% aqueous uranyl acetate (Serva, Germany). Specimens were embedded in epoxy resin (Serva, Germany). Ultrathin sections were prepared on an Ultracut-E Ultramicrotome (Reichert—Jung, Austria) or an Ultracut UC6 (Leica, Germany). Final staining was done with uranyl acetate (Serva, Germany) in methanol (Fisher Scientific, Germany) and lead citrate (Merck, Germany). Sections were analyzed with the JEOL JEM-1010 or the JEOL JEM-1400 plus transmission electron microscope (JEOL, Japan).

Lesions were quantified semi-quantitatively as follows: none scored = 0, then mild = 1, moderate = 2, and high grade = 3.

### Immunohistochemistry

Besides routine immunohistochemistry testing for IgG, IgM, IgA, C3, and C1q, ancillary testing for IgG4 and PLA2R was performed either originally or retrospectively, according to in-house protocols: 13/15 patients had at least one biopsy tested for IgG4 (two patients had insufficient material to conduct the staining), mostly retrospectively. Testing for PLA2R occurred for cases originally not tested for it and when prior testing for IgG4 was positive and for FAT-1 negative (7/15). Routine immunohistochemistry for IgG subclasses other than IgG4 is typically not part of standard procedures in most laboratories globally, so it was not conducted.

### Immunofluorescence from FFPE material

Cases with available material (*n* = 12) were stained with antibodies against FAT1 (FAT1 polyclonal antibody, rabbit, Invitrogen cat. no. PA5-51647) in a dilution of 1:1000. Slides were incubated at 56°C overnight, deparaffinized in Xylol and rehydrated in decreasing concentrations of ethyl alcohol. A decloaking chamber was used at 125°C for 5 min. The slides were then washed with PBS and blocked in 5% goat serum in 0.5% PBS/Tx100 for 30 minutes. The first antibody was incubated in blocking buffer at 4°C overnight, then washed with PBS followed by the incubation of the second antibody (Alexa Fluor 546, 1:1000) in blocking buffer for 1 hour at room temperature. The slides were then washed in PBS again, counterstained with DAPI and washed once more, then cover slipped. To validate our protocol, we also stained samples from colon, testis, cerebellum, and stomach (Supplementary Fig. 2).

Expression intensity (none = 0, low = 1, medium = 2, high = 3) and distribution were recorded.

Two cases (patients 10 and 13) did not have available FFPE material anymore. We decided to repurpose archived HE sections for immunofluorescence. We first tested feasibility on HE-stained control stomach biopsies that were cover slipped and kept for 2 weeks at room temperature, then incubated them in *n*-butyl acetate for as long as necessary for cover slip removal, de-stained them in 1% HCl-EtOH under regular microscopic control, and submitted them for FAT-1 immunofluorescence and reaching an acceptable result. The two kidney biopsy cases were submitted to this pretreatment and then to the same FAT1-immunofluorescence protocol as described before.

One patient had already been explored by mass spectrometry for FAT1 [11].

## Results

### Immunofluorescence for FAT1

**Figure 1:**
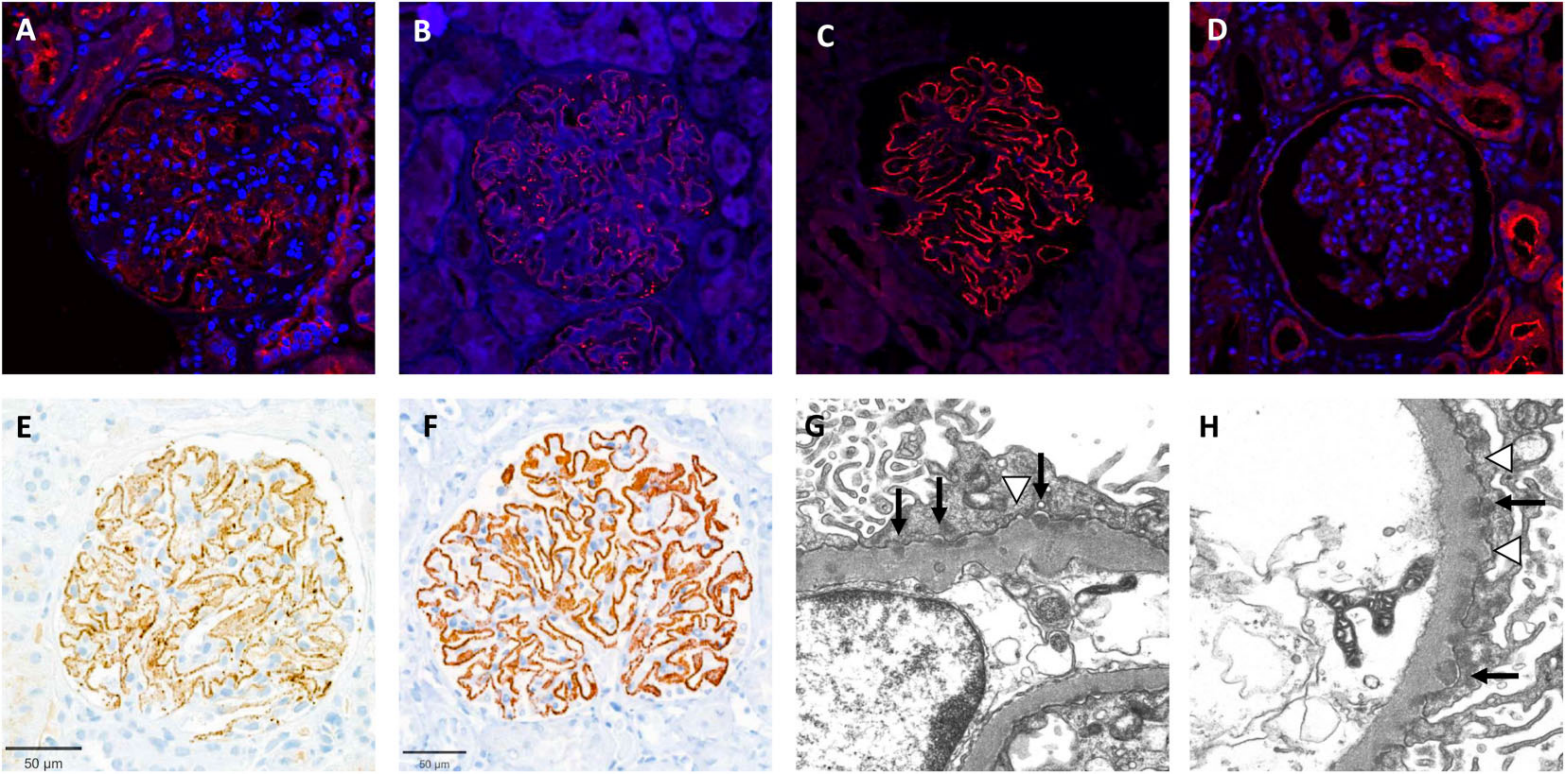
Immunofluorescence of FAT1 ranged from focal-segmental low intensity (A, case 1) to diffuse-global intermediate intensity (B, case 10) to diffuse-global marked intensity (C, case 13). (D) Control cases showed no signal on immunofluorescence. (E) Immunohistochemistry for IgG4 and (F) PLA2R was performed wherever needed: (E) and (F) belong to case 6 and show a strong global-diffuse granular signal. In addition, electron microscopy was performed where material was available. (G) and (H) Electron dense subepithelial depositions (black arrows) with concurrent basement membrane remodeling (white arrowheads).

Immunofluorescence on FFPE biopsies (and in one case mass spectrometry) revealed FAT1-positivity in 12 out of 15 patients, with an expression of 2 [interquartile range (IQR) 2–3] and a diffuse and global distribution in most cases (Fig. 1). Two patients had a repeat biopsy that was FAT1-positive (referred to as FAT1+ MN) again. The four control cases were all negative for FAT1 (referred to as FAT1− MN).

In the validation samples, FAT1 expression with our protocol was comparable to expression described in the human protein atlas and by the manufacturer of the diagnostic anti-FAT1 antibody (Invitrogen, PA5-51647) (Supplement Fig. 2).

### Histological and immunohistochemical findings

Three biopsies showed fewer than 10 vital glomeruli, but the number of vital glomeruli per biopsy was 15 (7–18.5) for FAT1+ MN and 21 (16.5–23.5) for FAT1− MN cases. Glomerular sclerosis was 13.4% (6.1–26) for FAT1+ MN and 19.2% (9.6–31) for FAT1− MN.

With regard to mesangial characteristics, both groups displayed mild to moderate mesangial expansion, with a of 1 (1–2), whereas only FAT1+ MN cases (n = 3) had mild to moderate mesangial hypercellularity. A total of 13 biopsies from both groups displayed lesions of the basement membrane (spikes, holes, double contours, or a combination of those) on light microscopy.

Interstitial fibrosis with tubular atrophy was negligible to mild in all FAT1+ MN cases [10% (2.5–37.5)], one case of FAT1− MN had extensive interstitial fibrosis and tubular atrophy, most probably due to additional unspecific interstitial nephritis.

Immunohistochemistry most commonly displayed depositions of IgG in a diffuse segmental granular distribution along the basement membranes of the glomeruli. Intensity of IgG deposits ranged from mild to strong and was 2 (1–3) in FAT1+ MN and 1 (1–1) in FAT1− MN (Fig. 1). Other immunoglobulins showed IgM signal intensity of 2 (1–2) FAT1+ MN and 0 (0–0) in FAT1− MN, no IgA deposits, and for complement C3 in 0 (0–1) in FAT1+ MN and 0.5 (0.25–0.75) in FAT1− MN, for C1q 2 (1–2) in FAT1+ MN and 0.5 (0.25–0.75) in FAT1− MN.

Staining for IgG subclass 4 was conducted at the time of routine biopsy, or retrospectively in 16 biopsies (13 cases): 11 were negative and five were positive, with no switch of reactivity between two biopsies in patients with repeated biopsies. In FAT1+ MN 3/12 (one with a repeat biopsy) were IgG4-positive, in FAT1− MN this was 1/3.

Eight cases were originally tested for PLA2R and one case was positive. Cases without original routine testing for PLA2R (*n* = 7)—most of the time before the identification of PLA2R as a putative causal antigen—were not retrospectively submitted to this test as these cases were all IgG4-negative and four of them were also FAT1-positive.

Of the three FAT1− MN, one was PLA2R-positive in immunohistochemistry, one was negatively tested for NELL1 (courtesy of Prof. Maike Büttner-Herold, Institute of Pathology, Department of Nephropathology, Erlangen, Germany), and one did not have material left for further testing.

The four control MN cases showed no expression of FAT1. The two PLA2R-positive MN control cases showed a strong expression of PLA2R and IgG4 in immunohistochemistry, and the two lupus nephritides cases showed a strong expression of IgG.

### Clinical presentation

Taking into account the first biopsy with MN for each patient, there were 8/12 males (67%) and 2/3 males (67%) among FAT1+ MN and FAT1− MN patients, respectively, with an average age of 53 (46.5–59.3) vs 45 years (27.5–51.5). Indication for biopsy was proteinuria in all but one biopsy. Out of 19 times, 15 of the patients presented with a nephrotic syndrome. FAT1+ MN vs FAT1− MN had a urinary protein-to-creatinine ratio (in g/g): 8 (4.6–9) vs 10.4 (9.7–11.1), respectively. Among the FAT1+ MN cases, five (42%) presented with additional hematuria.

Serum creatinine at time of biopsy was 1.2 mg/dl (0.8–1.3) for FAT1+ MN and 1.3 (1–1.6) for FAT1− MN patients (Table 1), one patient being on dialysis and excluded from this data (patient 9 at the time of first biopsy).

**Table 1:** Initial clinical data and antigen exploration.

Patient	Age (year)/sex	Serum creatinine (mg/100 mL)	Proteinuria in UPCR (g/g)	Indication for HSCT	PLA2R	IgG4	FAT1
1	59/F	0.78	Proteinuria 1.5	Aplastic anemia		negative	positive
2	39/F	n/a	Nephrotic syndrome 3.9	Chronic myeloblastic leukemia (+hepatitis C)		negative	positive
3	63/F	1.3	Nephrotic syndrome 9	Myelofibrosis secondary to polycythemia vera	negative	negative	positive
4	10/F	1.96	n/a	Chronic granulomatous disease caused by homozygotic mutation of NCF1	negative	negative	negative
5	55/M	0.8	Nephrotic syndrome 6.5	Myelofibrosis		negative	positive
6	45/M	0.61	Nephrotic syndrome 11.8	Acute lymphoblastic leukemia	positive	positive	negative
7	26/M	0.68	Nephrotic syndrome 8	Acute lymphoblastic leukemia		Negative	positive
8	50/M	1.24	Nephrotic syndrome 8.3	Chronic myeloblastic leukemia		negative	positive
9	58/M	7	Nephrotic syndrome 9	Acute myeloblastic leukemia		n/a	negative
10	51/F	0.87	Nephrotic syndrome 4.7	Secondary AML after Hodgkin lymphoma, recurrence of disease		n/a	positive
11	49/M	0.8	Nephrotic syndrome 4.4	Multiple myeloma		negative	positive
12	60/M	1.39	Nephrotic syndrome 9	Mantle cell lymphoma	negative	positive	FAT1+ was confirmed via MS
13	28/M	1.3	Nephrotic syndrome 19	Acute myeloblastic leukemia		n/a	positive
14	62/M	1.36	Nephrotic syndrome 18	Myelodysplastic syndrome (+hepatitis C)	negative	negative	positive
15	57/M	1.2	Nephrotic syndrome 3.6	Myelodysplastic syndrome		negative	positive

Abbreviations: FAT1: protocadherin FAT1; HSCT: hematopoietic stem cell transplantation; MS: mass spectrometry; PLA2R; phospholipase A2 receptor.

FAT1+ MN patients had MN occurring 2.3 years after HSCT (1.3–4.4) while FAT1− MN patients had it occurring after 3.8 years (2.5–17).

Reasons for bone marrow transplantation in FAT1+ MN patients were myelodysplastic syndrome (two: one patient in combination with hepatitis C), chronic myeloblastic leukemia (two: one patient in combination with hepatitis C), secondary acute myeloid leukemia after Hodgkin lymphoma, acute lymphocytic leukemia [2], primary myelofibrosis, secondary myelofibrosis following polycythemia vera, aplastic anemia, multiple myeloma and mantle cell lymphoma, and in the three FAT1− MN patients acute myeloid leukemia [2] or chronic granulomatous disease with NCF1 mutation (Table 1).

### Electron microscopy

Electron microscopy of glomeruli was conducted on 12 of the samples (12 patients, nine of whom had FAT1+ MN). All but two of the cases showed either subepithelial [7] or intramembranous [3] electron dense deposits. The two remaining cases showed electron lucent areas, one of them with marked thickening of the basement membrane (interpreted as resolving deposits) and one with additional subendothelial electron dense depositions. Podocyte foot process effacement was higher for FAT1+ MN, with a grade of 3 (2.5–3) and FAT1− MN 2 (1.5–2.5). Stage of MN according to Ehrenreich and Churg ranged from 1–4, with stage 2 being the most common one. There was no difference between FAT1+ and FAT− cases (median of 2 for both). All FAT1+ MN had at least mild mesangial expansion by EM.

### Treatment strategies, follow up and outcome data

Follow-up time for FAT1+ MN was 5.2 years (0.9–13.4), with the longest follow up being 25.1 years. These FAT1+ MN required a variety of immunosuppressive therapies. Of the 12 FAT1+ MN, patients had: one lasting spontaneous resolution: proteinuria was low from the beginning (1.5 g/g); one complete remission under steroid therapy only; one complete remission under cyclosporin-based therapy; four rituximab-based therapies: two complete and two partial responses; two rituximab- and cyclosporin-based therapies: one complete response and one refractory case; two cyclophosphamide-based therapies: one complete response (with cyclosporin and rituximab) and one refractory case (with cyclosporin, follow up of only 8 months); and one patient under steroid only and no response at 1 year: rituximab therapy is pending.

Leaving aside the cases with spontaneous resolution or pending rituximab therapy, complete or partial response was reached in 60% or 20%, respectively (Table 2). Under rituximab-based therapy (*n* = 7), four (57%) patients experienced complete remission, two (29%) partial remission, and one (14%) was refractory to treatment. Partial remission was defined as >50 reduction of initial albuminuria. Complete remission was defined as >90% reduction of initial albuminuria or <0.5 g albuminuria/day. Data on treatment response were not available for five patients.

**Table 2:** Initial and follow-up clinical data.

Patient	Initial creatinine (mg/dL)	Creatinine at 12 months (mg/dL)	Creatinine at 36 months (mg/dL)	Creatinine at 60 months (mg/dL)	Initial Proteinuria (UPCR in g/g)	Proteinuria at 12 months	Proteinuria at 36 months	Proteinuria at 60 months	Treatment	Therapy response	Follow up (years)
1	0.78	0.99	0.89	0.82	1.5	0.1	0	0	RPT	SR	15.16
2	1.19 (bx2)	n/a	1.75 (bx2)	1.82 (bx2)	3.9	n/a	0.3	0	Steroids	CR	9
3	1.3	1.3	n/a	n/a	9	n/a	n/a	n/a	RTX (2x)	PR	0.69
4	1.96	n/a	1.96	n/a	n/a	n/a	n/a	n/a	n/a	n/a	2.93
5	0.8	1.06	1.33	3.85	6.5	2	3.4	7	ACE inhibitors, Tac, RTX (2x)	NR	5.26 (death)
6	0.61	0.77	0.76	n/a	11.8	1	0.2	n/a	RTX (2x)	n/a	3.28
7	0.68	1.2	0.78	0.74	8	0.2	<0.1	<0.1	CyA, CP, steroids, RTX (4x)	CR	13.38
8	1.24	1.23	1.15	1.14	8.3	3	0.6	0.3	CyA, steroids	CR	5
9	7 (bx 1)	1.46	1.43	n/a	9	0.3	0.2	n/a	Dialysis	n/a	21.18
		(bx 5)	(bx 5)								
10	0.87	n/a	n/a	n/a	4.7	n/a	n/a	n/a	CyA, PP (for TMA), Tac, CP, steroids	NR	0.7 (death)
11	0.8	1.1	1.3	1	4.4	2.5	0.1	0.1	RTX, CyA followed by RPT	CR	13.24
12	1.39	n/a	n/a	n/a	9	2.8	n/a	n/a	RTX (2x)	CR	2.05
13	1.3	0.9	0.87	1.06	19	1.8	n/a	n/a	Steroids, RTX	CR	8.98
14	1.36	n/a	n/a	n/a	18	n/a	n/a	n/a	RTX (2x)	PR	0.2
15	1.2	1.19	n/a	n/a	3.6	6.4	n/a	n/a	Steroids, CyA, after diagnosis discontinuation of CyA, RPT or RTX (2x)	pending	0.86

Abbreviations: Bx1 or 2 or 5, first or second or fifth biopsy; Cya, cyclosporin A; CP, cyclophosphamide; NR, non-responder; PP, plasmapheresis; RPT, renoprotective therapy; RTX, rituximab; Tac, tacrolimus; SR, spontaneous remission; TMA, thrombotic microangiopathy.

The three FAT-MN cases had: one patient with complete remission under steroid therapy only; one patient with unknown outcome after rituximab therapy; and one patient with unknown therapy and outcome.

During follow up [FAT1+ MN 5.1 years (0.8–10) vs FAT1− MN 3.3 (3.1–12.2)], two FAT1+ MN patients died after MN diagnosis: one patient (patient 5) of cerebral edema after 6 years (HSCT due to myelofibrosis) and a second patient (patient 10) died after 8 months (HSCT for AML secondary to recurring Hodgkin lymphoma) with the cause of death unknown to us (Table 2).

## Discussion

FAT1 is one of the most recently discovered antigens responsible of MN, first described after bone marrow transplantation, then in the context of kidney allograft with *de novo* MN and antibody-mediated rejection [9–12]. In the context of HSCT, the original publication described a discovery cohort of nine patients FAT1+ MN from the Mayo Clinic in Rochester, the findings of which could be validated in a supplementary cohort of five patients with post-HSCT MN from Cedar Sinai in Los Angeles. Our cohort adds 12 observations to these, valuable thorough clinico-pathological data, new evidence on therapy options, and, partly limited information of long follow up (>5 years), due to the retrospective collection of data. Two of these cases required repeat biopsy, confirming FAT1-positivity and IgG subclass specificity. Hence, this study validates FAT1 as the major causal antigen for post-HSCT MN, compiling up to 80% of the cases in our cohort; the other causal antigens being PLA2R in 7% and unknown antigens in 13% (with a putative role of NELL1 in one case), as described earlier [14].

### Clinico-pathologically distinctive features of the present cohort and earlier descriptions

Clinical presentations between the cohort of Sethi *et al.* [9] and ours did not differ dramatically, with a comparable male-to-female ratio (USA 64%, Vienna 67% male), a slightly younger cohort [USA 62.5 years (54–68.8), Vienna 53 years (46.5–59.2)], and a comparable time to MN diagnosis after HSCT in years [USA 2 years (2–3), Vienna 2.3 years (1.3–4)]. A notable difference resided in the diseases leading to HSCT, as the original cohort had mostly AML cases (57%), while ours displayed various disorders, although hematologic neoplasia dominated. Those distinctions could be related to different therapeutic strategies incorporating HSCT.

Serum creatinine levels at diagnosis were comparable to ours [USA 1.2 mg/dL (1.1–1.4)], but our cohort had higher levels of proteinuria [USA 5.6 g/24 h (3.9–8.5), if we accept the rough equivalence of 1:1 with our urine protein-to-creatinine ratio units (g/g and g/24 h)]. Concerning MN-specific treatment, both populations reached complete response after therapy with the same frequency (6/11). Our cohort with its complete therapy history may hint toward satisfactory results with rituximab (alone or in combination) leading to four complete and two partial remissions in seven patients. Last, the high AML frequency in the US cohort—a rather aggressive disease—could explain a higher rate of reported death.

Our pathological findings provide, for the first time, a thorough description of glomerular findings, with a non-negligeable (25%) portion of cases displaying features of what was earlier called “atypical” MN, including mesangial sclerosis and hypercellularity. As our immunohistochemical stains on FFPE for routine workup cannot, by nature, be compared to results of immunofluorescence on frozen material of the original description, a direct comparison is not feasible. In addition, as IgG subclasses testing for IgG1, IgG2, and IgG3 are not routinely available in most renal pathology laboratories using diagnostic immunohistochemistry, we cannot provide this valuable data. However, electron microscopy findings summarized with the MN stages according to Ehrenreich and Churg are comparable with identical median stages of 2, in spite of some more advanced cases—one stage 3 and three stage 4–in the latter cohort.

### FAT1 immunofluorescence on FFPE is a feasible approach

Our study demonstrates that FAT1 immunofluorescence is a feasible tool to identify FAT1+ MN on FFPE material. We found that our protocol allowed convincing and reliable staining results. To our knowledge FAT1 immunohistochemistry on FFPE may prove challenging and frozen tissue is not available in many pathology departments. We therefore implemented the immunofluorescence on FFPE. It allowed to combine IF as sensitive technique to a better morphology provided by FFPE material.

### Pathophysiology

From a pathophysiologic point of view, the biologic role of FAT cadherins is not well understood. However, FAT1 is localized at cell-to-cell junctions in podocytes and loss of FAT1 results in podocyte effacement and steroid resistant nephrotic syndrome [15–17]. Interestingly, most cases with FAT1-associated MN reported until now are patients after HSCT, followed by some *de novo* allograft MN associated with antibody-mediated rejection. This raises the suspicion of a distinct pattern of glomerular damage rather specific to the HSCT procedure, leading to increased or aberrant expression of FAT1 in glomerular cells (possibly podocytes), rendering FAT1 accessible to recognition by immune cells. We speculate that HSCT conditioning regimens, typically including high-dose chemotherapy and whole-body irradiation, could provide this rather specific glomerular damage. Following immune reconstitution, a process associated with dysregulated B-cell recovery and B-cell activating factor (BAFF) environment, prolonged B-cell lymphopenia with high BAFF favoring survival, and expansion of autoreactive B-cell clones that would normally be deleted, could promote the production of new pathogenic auto- and alloantibodies [18, 19]. As such, cGVHD reflects ongoing donor immune activity and is frequently associated with secondary autoimmune phenomena. cGVHD and the general alloimmune environment can promote production of antibodies that cross-react with host tissues, including kidney antigens [20].

In the case of FAT1 as a target antigen, MN patient sera/biopsy-eluted IgG can bind FAT1 [9]. That identifies a mechanistic link where newly produced antibodies during immune reconstitution bind podocyte-associated FAT1, build subepithelial immune complexes, and result in MN. Furthermore, Sethi and colleagues hypothesized that a somatic/previously hidden FAT1 mutation may cause an immune response in the setting of GVHD [9]. A last aspect of this conundrum comes from the variably detectable IgG subclasses in FAT1+ MN, as originally described by Sethi *et al*. and confirmed in our cohort with a low frequency of IgG4 detection. The immune reconstitution and immunological disturbances (in patients with often malignant diseases leading to HSCT) are the basis of a complex interplay leading to antigen recognition and IgG subclass selection, not fully elucidated yet [21].

### Clinical implications

Our findings support routine immunostaining for FAT1 in HSCT patients with MN. Based on our hypothesis of *de novo* formation of anti-FAT1 antibodies in such cases, the development of an assay, possibly an ELISA, to detect circulating anti-FAT1 would be the next logical step.

Testing for circulating anti-FAT1 antibodies would greatly improve the diagnostic armamentarium of patients with proteinuria after HSCT. Such an anti-FAT1 antibody ELISA could be added to the current standard-of-care diagnostic panel of anti-PLA2R-antibodies and anti-THSD7A-antibody detection. Furthermore, anti-FAT1-antibody levels might serve as response monitoring after immunosuppressive treatment, such as rituximab, and guide the dose and duration of such therapies. In addition, early detection of circulating anti-FAT1-antibody levels could potentially be used to detect immunological relapses, even before recurrence of proteinuria.

Currently, there is no HSCT-specific randomized trial, but reported management follows MN and GVHD principles: optimize conservative therapy (ACEi/ARB, diuretics, anticoagulation when indicated), treat the underlying immune driver (control GVHD, reduce/adjust calcineurin inhibitors if implicated) and use immunomodulation for antibody-mediated disease (steroids, calcineurin inhibitors, mycophenolate, and B-cell depletion such as rituximab). Decisions must balance immunosuppression versus infection risk in the post-HSCT setting.

### Limitations

This study was limited by the cohort size. It is large in comparison to other cohorts describing this entity, but nonetheless small in terms of absolute numbers, which severely limits statistical analyses. It was also limited by the nature of our specialized center, which functions as a reference center for the entire country, leading to some patients being lost to follow up/having incomplete follow-up data. Furthermore, we could only test one case (P9) for another potential antigen, NELL1, which was negative, and insufficient tissue precluded further *in situ* testing in the other case without a determinable antigen. The only IgG subclass we tested for was IgG4. Last, we did not test for the presence of anti-FAT1 antibodies in patients’ sera.

## Conclusion

With this cohort, we add 12 more patients to the growing list of cases with FAT1+ MN post-HSCT. This study illustrates that FAT1 immunofluorescence is a feasible tool to identify FAT1+ MN on FFPE material. Not all patients with MN post-HSCT showed positivity for FAT1, suggesting further antigens and disease mechanisms to explore.

## Supplementary Material

sfag005_Supplemental_Files

## Data Availability

The data underlying this article will be shared on reasonable request to the corresponding author.
