# Supplementary figures and images for: Protocadherin FAT1-associated membranous nephropathy after hematopoietic stem cell transplantation—a cohort study with clinico-pathological correlations
Source: Clin Kidney J. 2026 Jan 10;19(3):sfag005. doi: 10.1093/ckj/sfag005 (PMC13098137; doi:10.1093/ckj/sfag005)

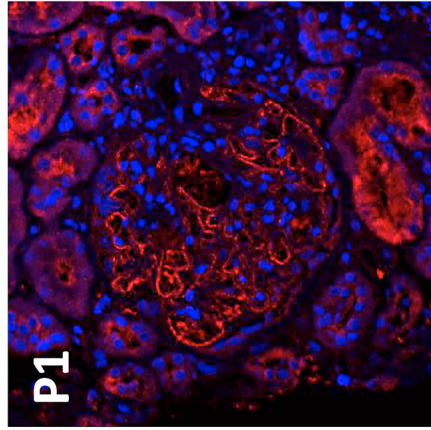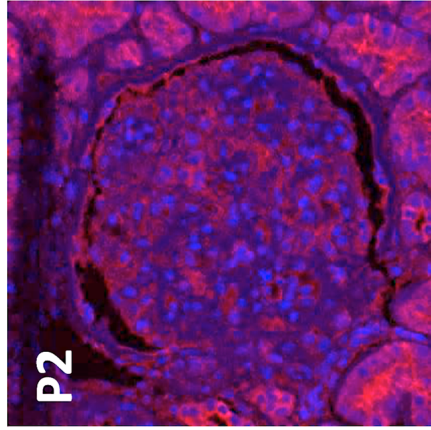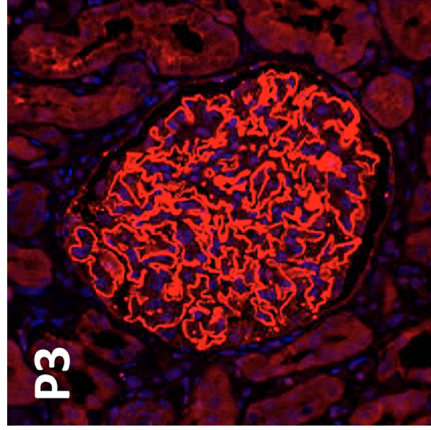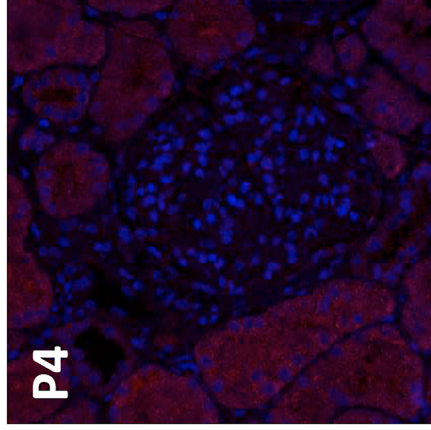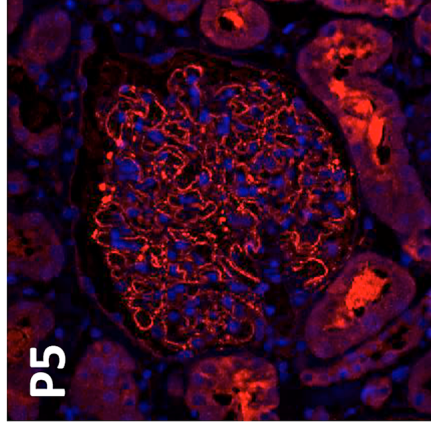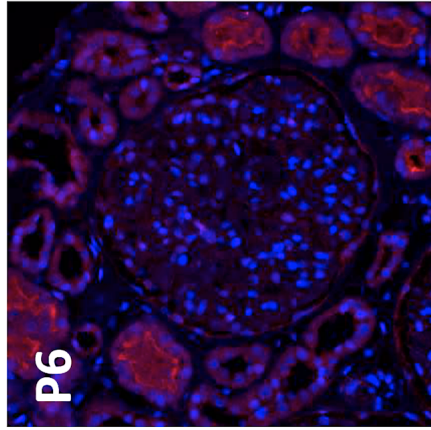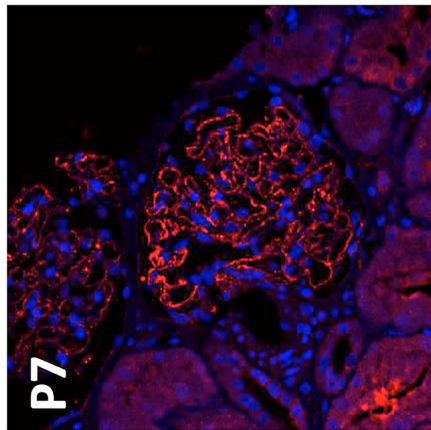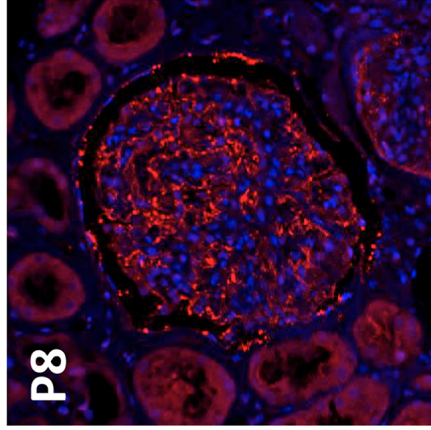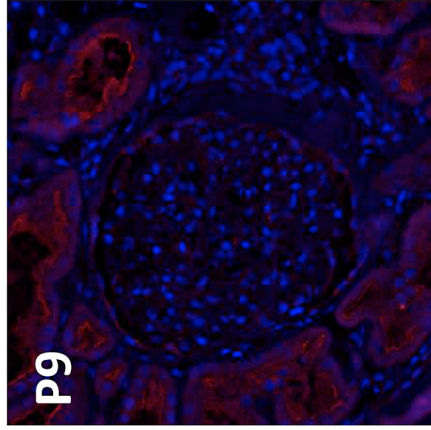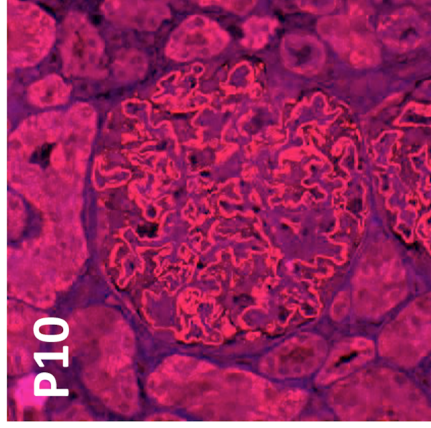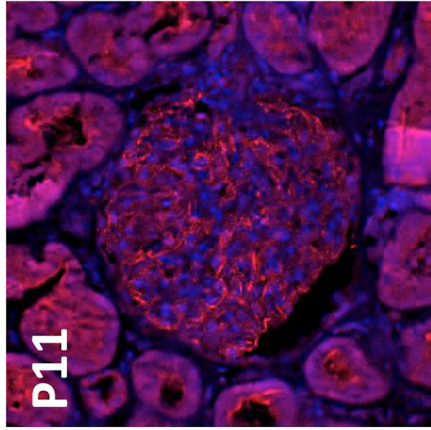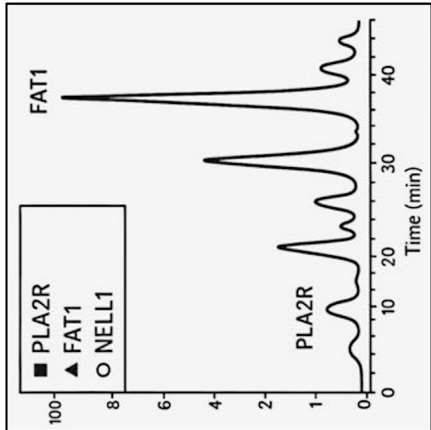

Supplement: sfag005_Supplemental_Files [file sfag005_Supplemental_Files.zip › FAT1_supfig1.pdf]

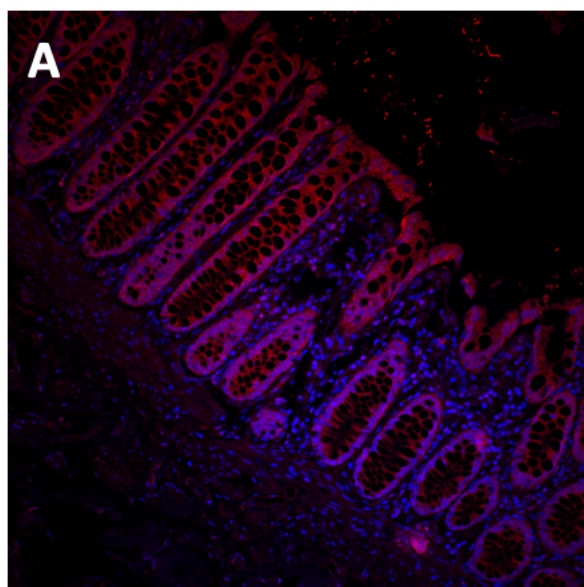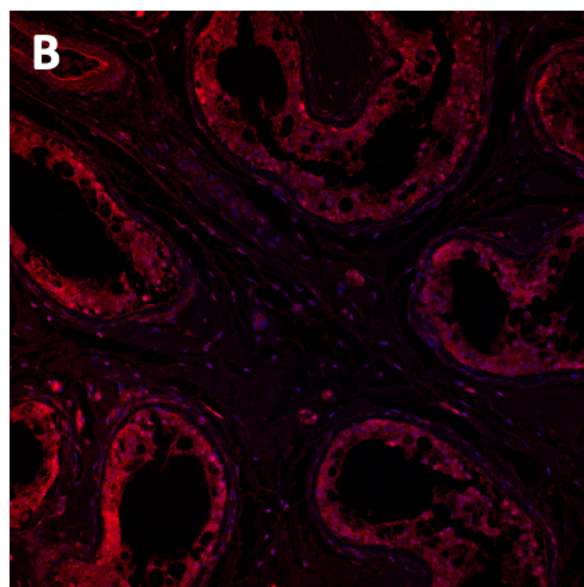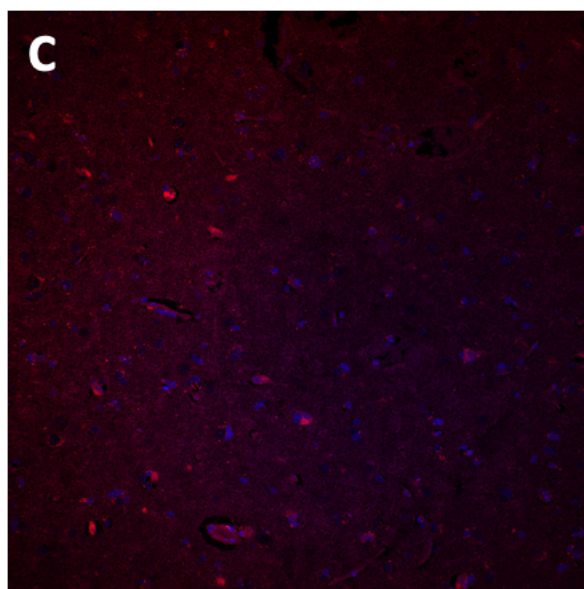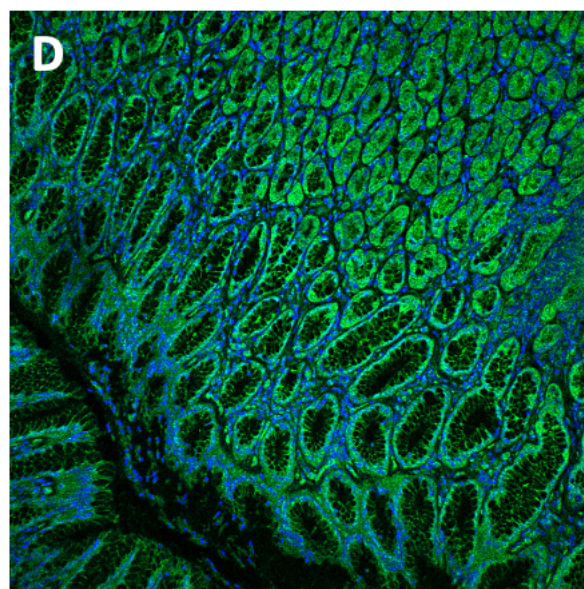

Supplement: sfag005_Supplemental_Files [file sfag005_Supplemental_Files.zip › FAT1_supfig2.pdf]
